# Assessment of Health-Related Quality of Life in Chronic Kidney Disease Patients: A Hospital-Based Cross-Sectional Study

**DOI:** 10.3390/medicina59101788

**Published:** 2023-10-08

**Authors:** Shivam Sharma, Darpan Kalra, Ishfaq Rashid, Sudhir Mehta, Manish Kumar Maity, Khushi Wazir, Sumeet Gupta, Siddique Akber Ansari, Obaid S. Alruqi, Roohi Khan, Imran Khan, Sirajudheen Anwar

**Affiliations:** 1 Department of Pharmacy Practice, M.M. College of Pharmacy, Maharishi Markandeshwar University, Ambala 133207, India; shivammoudgil33@gmail.com (S.S.); darpankalra0001@gmail.com (D.K.);; 2 Department of Pharmacotherapy, College of Pharmacy, University of Utah, 30S 2000E, Salt Lake City, UT 84112, USA; 3 Department of Nephrology, M.M. Institute of Medical Sciences and Research, Maharishi Markandeshwar University, Ambala 133207, India; 4 Department of Pharmacology, M.M. College of Pharmacy, Maharishi Markandeshwar University, Ambala 133207, India; sumeetgupta25@mmumullana.org; 5 Department of Pharmaceutical Chemistry, College of Pharmacy, King Saud University, Riyadh 11451, Saudi Arabia; sansari@ksu.edu.sa (S.A.A.);; 6 Department of General Medicine, King Khaled Hospital, Hail 55421, Saudi Arabia; 7 Department of Pharmacology and Toxicology, College of Pharmacy, University of Hail, Hail 55476, Saudi Arabia

**Keywords:** chronic kidney disease, health-related quality of life, KDQoL

## Abstract

*Background:* Health-related quality of life is rapidly becoming recognized as an important indicator of how a disease affects patient lives and for evaluating the quality of care, especially for chronic conditions such as chronic kidney disease (CKD). *Objectives:* This study is an attempt to assess the quality of life in patients with chronic kidney disease at MMIMSR and also identify characteristics that may be associated with their worsening quality of life. *Materials and Methods:* This cross-sectional investigation was conducted at the in-patient department (IPD) of the MMIMSR hospital. This study included 105 CKD patients and used a systematic random sampling method for quantitative analysis. This study utilized a 36-item short-form SF-36 (v1.3) questionnaire to assess HRQoL in CKD patients. Descriptive statistics were employed at the baseline. Chi square and ANOVA were used to draw comparisons between two groups or more than two groups, respectively. Logistic regression analysis was utilized to identify the potential QoL determinants. A *p* value of 0.05 or lower was used to determine statistical significance. *Results:* Among a total of 105 participants, the mean (±standard deviation) age was found to be 54.53 ± 13.47 years; 48 were male patients, and 57 were female patients. Diabetes Mellitus (61.9%), hypertension (56.2%), chronic glomerulonephritis (7.6%), chronic pyelonephritis (6.7%), and polycystic kidney disease (5.7%) were identified to be the most frequent disorders associated with CKD. The current study also demonstrated that the HRQoL score domains such as symptom problem list, the effect of kidney disease, and the burden of kidney disease decline significantly and progressively as the patient advances into higher stages of CKD (*p* = 0.005). A similar pattern was observed in work status, sleep, and general health (*p* < 0.005). Additionally, a statistically significant difference was noted for cognitive function, quality of social interaction, overall health, dialysis staff encouragement, patient satisfaction, social support, physical functioning, role of physical health, pain, emotional well-being, role of emotional health, social functioning, and energy fatigue (*p* < 0.005). The mean difference for PCS and MCS based on CKD stages was found to be statistically significant (*p* < 0.005). The PCS and MCS showed a positive correlation with GFR (r = 0.521), and Hb (r = 0.378), GFR (r = 0.836), and Hb (r = 0.488), respectively. *Conclusions:* The findings of this study demonstrated that a significant decrease in HRQoL was observed among CKD patients, with a progressive deterioration of HRQoL dimensions as the patient advances to end-stage renal disease. This study also revealed that CKD imposes various restrictions on patients’ day-to-day lives, particularly in terms of their physical and mental functioning, even in the initial stages of the disease.

## 1. Introduction

Chronic kidney disease (CKD) is a diverse condition where kidney function is reduced at various levels, starting from a state of risk or damage and progressing through mild, moderate, and severe stages of chronic kidney failure [1]. CKD has grown to be an enormous burden on the world’s healthcare system [2] and is now understood to pose a serious risk to people’s quality of life (QOL) as the condition advances [3]. Over 800 million people globally, or 10% of the overall population, are afflicted by chronic kidney disease [4]. CKD is linked to cardiovascular morbidity and death; it also impairs quality of life. Cardiovascular disease mortality has been anticipated as eight-to-ten-fold higher in CKD patients than in non-CKD persons [5]. The expenses of managing CKD co-morbidities are substantial, offering significant challenges to healthcare systems, particularly in low-income nations [6].

Patients with chronic kidney disease (CKD), including those with end-stage renal disease (ESRD), should pay close attention to their functional status and subjective well-being as they relate to their health condition, which are collectively known as health-related quality of life (HRQoL) measurements. The timing of starting dialysis, whether a patient continues to work despite developing kidney failure, or whether they continue to play an active role in their home and community, are all influenced by the health-related quality of life (HRQoL) of these patients.

The quality of life (QoL) of CKD patients, particularly those with end-stage renal disease (ESRD), is a distinct risk factor for mortality [7,8]. Additionally, numerous factors, including symptoms associated with the condition, adverse reactions to medications, and the degree to which patients interact with their families might affect quality of life [9,10]. Patients with CKD have a lower QoL, more symptoms, and greater psychological distress, and the degree of these changes is adversely linked with GFR [9,11]. End-stage renal disease patients scored lower on the HRQoL scale than the general population [12]. Low HRQoL scores among ESRD patients are correlated with both their socioeconomic and medical circumstances. The KDOQI guideline suggests routine measurements to evaluate the quality of treatment provided to hemodialysis patients and recognizes HRQoL as a fundamental outcome [13].

Low QoL has been the main issue for CKD patients, and its emergence can have a negative effect on CKD progression. Patients with CKD have lower QoL, more symptoms, and greater psychological distress, and the degree of these changes is adversely linked with GFR [11]. There is a dearth of information on the quality of life (QoL) of patients receiving conservative care and the connection between QoL and GFR. Some research has shown that QoL is decreased in the early stages of disease, despite the fact that individuals with advanced renal insufficiency had a reduced QoL.

In India, the prevalence of CKD has been recorded as 17.2%, of which 6% have stage 3 or more severe CKD [14]. Chronic kidney disease is becoming more common as a result of a rise in the prevalence of risk factors including diabetes and hypertension. To enhance QoL, healthcare practitioners in renal clinics would benefit from a comprehensive examination of variables impacting the QOL. Unfortunately, more evidence-based research is required to assess QoL and associated factors in patients with CKD from developing nations. This is especially crucial in resource-constrained nations such as India.

Against this backdrop, this study aimed to evaluate the quality of life and identify factors that might be contributing to the declining QoL in CKD patients at a public teaching hospital.

## 2. Materials and Methods

This cross-sectional study comprised 123 adult patients with chronic kidney disease. Eighteen (18) patients were excluded from the study. The reasons were incomplete biochemical analyses (10 patients), refusal to participate (4 patients), refusal to answer all the questions (3 patients), and severe dementia (1 patient). Participants who were under the age of 18 or over 80 years, pregnant, had kidney transplants, used drugs excessively, or had a history of cancer were also excluded from the study.

A total of 105 adult CKD patients were evaluated for HRQoL by using the 36-item SF-36 (v1.3) questionnaire at the MMIMSR hospital. This tool has been validated on CKD patients undergoing dialysis [15]. Each participant gave their written informed consent to take part in the study, and the institution’s ethics committee approved the protocol.

### 2.1. Study Procedure

The sociodemographic details and biochemical parameters (GFR, Hb, serum creatinine, sodium, total protein, chloride, potassium, albumin, serum uric acid and urea) were recorded at the baseline. Furthermore, GFR was calculated using the CKD EPI Equation. Body weight and height measurements were obtained, and the BMI (Body Mass Index) was computed.

Patients who attended an IPD at the nephrology unit were requested to complete the SF-36. Several patients were unable to complete the SF-36 (v1.3) on their own; their responses were recorded by the research investigator via proper consultation with the patients. A self-administered tool called the KDQoL SF-36 (v1.3) questionnaire was employed to assess general Health-Related Quality of Life, which is not illness- or treatment-specific. A higher score denotes a better-perceived health status, with a 100-point scale used to evaluate each question in the questionnaire. The KDQoL-36™ (version 1) is a short form that comprises the SF-12 (12 items) as generic core plus the burden of kidney disease (4 items), symptoms/problems of kidney disease (12 items), and effects of kidney disease (8 items).

In this study, we have utilized KDQoL SF-36 (1.3) (Table 1). The whole questionnaire is included as a supplement (Supplementary Materials). The physical dimensions of the symptom problem list, including the effect of kidney disease, the burden of kidney disease, sexual function, sleep, work status, overall health, and pain are typically combined to generate a physical composite summary (PCS). Additionally, the mental dimensions of cognitive function, the role of emotional health, patient satisfaction, quality of social interaction, social support, dialysis staff encouragement, emotional well-being, and social function are generally combined to generate a mental composite summary (MCS).

The SF-36 questionnaire is widely utilized and accepted in a wide range of contexts, enabling comparisons both within and between conditions. The recommendations of the National Kidney Foundation stress the need for the questionnaire to be reliable and accurate.

### 2.2. KDQoL SF-36 Scoring Rules

When applying the KDQoL-SFTM scoring process, which comprises translating pre-coded numeric values of responses to a range of 0–100, higher scores reflect a greater quality of life. This range is modified to meet the raw values of each item, with 0 being the lowest possible score and 100 denoting the greatest. Item 23, which has a pre-coded range of 1 to 7, is recorded by subtracting the raw value by 1, dividing the difference by 6, and then multiplying the outcome by 100. The sexual function scale’s item 16 is crucial; if the answer is “no”, the scale’s score should be regarded as missing. The scores for each scale are determined by averaging their items.

### 2.3. Ethical Considerations

The institutional ethics committee thoroughly reviewed the research proposal, assessing factors such as informed consent procedures, participant privacy and confidentiality, potential risks and benefits, and compliance with relevant laws and regulations. Patient’s rights were respected and maintained. The concerned Institutional Ethics Committee has approved this study [Reference number: 2333].

### 2.4. Statistical Analysis

Descriptive statistics including frequency, percentages, mean, and standard deviation were used to summarize the baseline sociodemographic data and clinical characteristics of the patients. For categorical data, the chi-square test was used, and one-way ANOVA was used to assess the continuous variables based on the stages of CKD. Pearson correlation was utilized to evaluate the relationship between dimensions of SF-36 and different covariates. Statistics are deemed significant with *p* values under 0.05. Data were organized, cleaned up, coded, and entered into Microsoft Excel before being analyzed with SPSS version 20.0, Chicago (IL, USA).

## 3. Results

### 3.1. Participants and Characteristics

Among a total of 105 participants, the mean (±standard deviation) age was found to be 54.53 ± 13.47 years; 48 were male patients, and 57 were female patients. The study had a higher proportion of adult patients (<60 years) (*n* = 63) as compared to elderly patients (≥60 years) (*n* = 42). A 38.1% prevalence of hyperuricemia was reported, with CKD stage 5 patients accounting for the largest portion (21.9%). A statistically significant difference was observed for CKD stages based on hyperuricemia (*p* = 0.02) (Table 2).

CKD stage 5 patients had a higher mean age (55.07 ± 13.45) as compared to CKD stage 3 patients (51.83 ± 14.87). Regarding the other laboratory findings, CKD stage 5 patients reported higher baseline creatinine levels, lower hemoglobin levels, and higher serum uric acid levels. The urea levels were extremely high in patients with CKD stage 5 (164.26 ± 82.01) as compared to CKD stage 4 (87.70 ± 39.26) and CKD stage 3 (57.54 ± 41.06) (Table 3). The mean baseline estimated GFR was found to be 21.01 ± 16.70 mL/min/1.73 m^2^.

### 3.2. Complications Associated with CKD Patients

**Figure 1:**
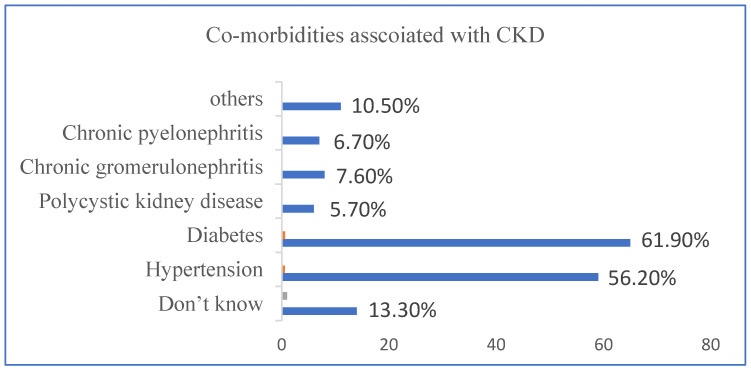
This figure provides a detailed overview of with and without co-morbidities associated with CKD.

Diabetes Mellitus (61.9%), hypertension (56.2%), chronic glomerulonephritis (7.6%), chronic pyelonephritis (6.7%), and polycystic kidney disease (5.7%) were identified to be the most frequent complications in this study. Other less-common etiologies include miscellaneous and obstructive uropathy (Figure 1).

Both diabetes and hypertension were present in 39.04% of the patients. Whereas, 1.90% of patients also had chronic glomerulonephritis or chronic pyelonephritis in addition to diabetes and hypertension. In 2.85% of patients, obstructive nephron disease and hypertension were related to other etiologies. An unspecified, other, non-identified, and other cause was recorded in 20% of patients. In 6.66% of patients, chronic pyelonephritis, polycystic kidney disease, and chronic glomerulonephritis are associated with hypertension. In 3.80% of patients, chronic pyelonephritis, polycystic kidney disease, and chronic glomerulonephritis were shown to be present with diabetes.

### 3.3. Evaluation of Health-Related Quality of Life in Patients with CKD

The results demonstrated that the HRQoL scores in all dimensions of the scoring manual are progressively impaired. Table 4 shows the HRQoL dimension scores at various CKD stages. Compared to patients in CKD stages 4 and 5, patients in CKD stage 3 (*p* <0.05) had higher scores across all SF-36 domains comprising the physical and mental component summaries. The mean difference for PCS and MCS based on CKD stages was reported to be statistically significant (*p* < 0.005) (Table 4).

The current study also demonstrated that the HRQoL score domains such as symptom problem list, the effect of kidney disease, and the burden of kidney disease decline significantly and progressively as the patient advances into higher stages of CKD (*p* < 0.005). A similar pattern was observed in work status, sleep, and general health (*p* < 0.005).

Additionally, a statistically significant difference was identified in cognitive function, social support, emotional well-being, quality of dialysis staff encouragement, social functioning, overall health, patient satisfaction, the role of physical health, physical functioning, pain, the role of emotional health, social interaction, and energy fatigue (*p* < 0.005). Additionally, this study examined the associations between various variables and the physical composite summary and the mental composite summary of the HRQoL tool (Table 5).

The Table 5 represents the correlation between age, GFR, Hb, serum creatinine, sodium, total protein, chloride, potassium, albumin, uric acid, urea, and the physical composite summary. A statistically significant correlation was observed for GFR, Hb, serum creatinine, total protein, chloride, and urea (*p* < 0.05). The PCS had shown positive correlation with GFR (r = 0.521, *p* < 0.005) and Hb (r = 0.378, *p* < 0.005), while a negative correlation was observed for creatinine (r = −0.665, *p* < 0.005), sodium(r = −0.140), total protein (r = −0.257, *p* < 0.005), chloride (r = −0.256, *p* < 0.005), potassium (r = −0.066, *p* = 0.008), albumin (r = −0.167, *p* = 0.009), uric acid (r = −0.117, *p* = 0.236), and urea (r = −0.450, *p* < 0.005).

Table 6 represents the correlation between age, GFR, Hb, serum creatinine, sodium, total protein, chloride, potassium, albumin, uric acid, urea, and the mental composite summary. A statistically significant correlation was observed for GFR, Hb, serum creatinine, total protein, albumin, and urea (*p* < 0.05). The MCS had shown a positive correlation with GFR (r = 0.836, *p* < 0.005) and hemoglobin (r = 0.488, *p* < 0.005). However, a negative correlation was identified with serum creatinine (r = −0.769, *p* < 0.005), total protein (r = −0.305, *p* = 0.002), albumin (r = −0.279, *p* = 0.004), and urea (r = −0.640, *p* = 0.004) (Table 6).

#### Effect of Quality-of-Life Domains (PCS and MCS) on CKD Associated Diabetes and Hypertension

A logistic regression was performed to ascertain the effects of PCS, and MCS on the CKD-associated disorders including diabetes and hypertension. The model successfully identified 62.9% of cases with CKD-associated diabetes and explained 10.0% (Nagelkerke R2) of the variation in those cases. Increasing PCS (β = −0.038; *p* = 0.240) and MCS (β = −0.032; *p* = 0.203) was associated with decrease in CKD associated diabetes; however, the results were not statistically significant. In case of CKD-associated hypertension, the model explained 22.5% (Nagelkerke R2) of the variance and correctly classified 70.5% of cases. Increasing PCS (β = −0.031; *p* = 0.353) and MCS (β = −0.068; *p* = 0.011) were associated with decrease in CKD-associated diabetes; however, only MCS showed a statistically significant association.

## 4. Discussion

Health-related quality of life is a constantly increasingly relevant measure in assessing the effectiveness of chronic illness therapy, particularly in patients with advanced CKD. Patients’ subjective assessments of the disease play a critical role in determining medical decisions that take into account their physical, social, and emotional requirements. Various factors contribute to this decline in HRQoL, including inadequate nutrition, anemia, cognitive impairment, depression, sleep disorders, apathy, reduced physical and sexual functioning, and co-morbidities such as diabetes, hypertension, and cardiovascular diseases. Until now, HRQoL has only been considered as a consequence of an individual’s illness. However, HRQoL is gaining significance as a patient-centered measure and is acknowledged as a health system indicator. Despite this knowledge, little is currently understood about how kidney disease specifically impacts HRQoL and whether HRQoL predictors can be targeted for potential interventions.

In this study, we have investigated health-related quality of life, and evaluated factors that might be contributing to declining QoL in patients with CKD at a public teaching hospital. According to the study’s findings, CKD patients had a clinically significant decline in HRQoL, with a progressive worsening of HRQoL dimensions as the patient progressed to ESRD. This study also showed that, even in the initial stages of the disease, CKD imposes a number of limitations on patients’ day-to-day lives, particularly in terms of their physical and mental functionality. These findings were supported by Pei et al., who also highlighted that HRQoL is significantly diminished in CKD patients, and this decline serves as a predictor of future mortality [16].

The results also highlighted that a strong association was observed for the variables such as CKD stages, age, sex, hyperuricemia, and anemia with HRQoL ratings. Factors such as GFR, serum creatinine, total protein, hemoglobin levels, were discovered to have a statistically significant difference on HRQoL ratings based on CKD stages. Numerous studies reported that clinical and demographic variables including age [17,18], gender [19], concomitant conditions such as diabetes [17], anemia [20], and residual renal function [21] all have an impact on HRQoL.

Anemia is quite common in CKD patients and is linked to poor clinical outcomes [22]. In the current study, anemia was associated with a worsening of HRQoL measures. These findings were corroborated by Finkelstein et al., who also noted that anemia in CKS patients is linked to a lower quality of life in terms of health (HRQoL) [20]. Furthermore, numerous studies have demonstrated that erythropoietin-stimulating agent (ESA) therapy for anemia in CKD improves quality of life in terms of health [23,24,25].

HRQoL is significantly impacted by CKD, with the physical domains presenting the greatest challenges. This study also evaluated the HRQoL domains based on CKD stages.

In the present study, the physical composite summary had shown a positive correlation with GFR and Hb, while a negative correlation was observed for creatinine, sodium, total protein, chloride, potassium, albumin, uric acid, and urea. However, the mental composite summary reported a positive correlation with GFR and hemoglobin and a negative correlation was identified with serum creatinine, total protein, albumin, and urea. This was supported by the findings reported by Pagels et al. [26] and Aggarwal et al. [27]. Additionally, the results showed that increasing PCS and MCS values are related to a decline in CKD-associated disorders; however, the findings were not statistically significant. The small sample size could be the cause.

In addition to this, HRQoL scores deteriorated gradually and significantly as eGFR dropped, with CKD stage 5 showing the most severe impairment. This suggests a link between declining kidney function (as determined by eGFR) and deteriorating HRQoL across all parameters. The physical composite summary scores were more deteriorated than mental composite summary scores but both had a significant difference. These findings were in consonance with the results reported by Aggarwal et al. [27].

Diabetes Mellitus (61.9%), hypertension (56.2%), chronic glomerulonephritis (7.6%), chronic pyelonephritis (6.7%), and polycystic kidney disease (5.7%) were identified to be the most frequent complications in this study. Other less-common etiologies include miscellaneous and obstructive uropathy. It is simple to overlook the reality that for patients, maintaining their mental health and being satisfied with their care are equally if not more critical than achieving clinical or quantitative laboratory targets. Here, we found that patients with CKD, particularly those in the final stages of the disease, had decreased HRQoL, a critical predictor of patient-centered outcomes, proving that quality of life is significant for both positive and negative outcomes for these patients.

Limitations of the study: The study’s cross-sectional design precluded a follow-up, which would have allowed for a better design for detecting the worse quality of life and underlying causes. Additionally, due to the data’s quantitative structure, the causes for the patients’ poor quality of life could not be adequately highlighted. These reasons could have been better understood by conducting in-depth interviews or focus groups. Furthermore, as the study concentrated only at a single facility and used a smaller sample size, caution should be used when extrapolating the findings.

## 5. Conclusions

This study found a significant decline in Health-Related Quality of Life (HRQoL) among chronic kidney disease (CKD) patients, with worsening HRQoL dimensions as the disease progresses to end-stage renal disease. CKD places restrictions on patients’ daily lives, particularly in physical and mental functioning, even in the early stages. Patients must actively manage their condition and maintain optimism for improved Quality of Life (QoL). The disease’s impact extends to the patients’ families, who also require ongoing information and support. Healthcare professionals should be aware of these effects and provide guidance for better daily living. Emphasis should be placed on psychosocial and medical therapies to enhance QoL in CKD patients. Despite limitations including a small sample size and single-center investigation, this study highlights the link between CKD and HRQoL, emphasizing the importance of assessing HRQoL in CKD patients. Timely interventions to improve HRQoL can significantly benefit patient health.

## Figures and Tables

**Table 1:** Items included in KDQoL SF-36 (1.3).

Scale	Number of Items	Specific Items Included
ESRD-Targeted Areas		
Symptom problem list	12	14a–k (l,m) *
Effects of kidney disease	8	15a–h
The burden of kidney disease	4	12a–d
Work status	2	20,21
Cognitive function	3	13b,d,f
Quality of social interaction	3	13a,c,e
Sexual function	2	16a,b
Sleep	4	17,18a–c
Social support	2	19a,b
Dialysis staff encouragement	2	24a,b
Patient satisfaction	1	23
36-item health survey		
Physical functioning	10	13a–j
Role physical	4	4a–d
Pain	2	7,8
General health	5	1,11a–d
Emotional well being	5	9b,c,d,f,h
Role of emotional health	3	5a–c
Social function	2	6,10
Energy/Fatigue	4	9a,e,g,i

* KDQoL SF 36 domains. ESRD: End-Stage Renal Disease; KDQoL-SF: Kidney Disease Quality of Life Short-Form.

**Table 2:** Distribution of baseline demographic characteristics based on CKD stages.

Variables	*n* (%)	CKD Stage 3 31(29.5%)	CKD Stage 4 20(19.0%)	CKD Stage 5 54(51.4%)	*p* Value
Gender					
Male	48(45.7)	14(13.3)	10(9.5)	24(22.9)	0.911
Female	57(54.3)	17(16.2)	10(9.5)	30(28.6)	
Age group					
Adults	63(60)	19 (18.1)	12 (11.4)	32 (19.5)	0.983
Elderly	42(40)	12(11.4)	8(7.6)	22(21.0)	
Hyperuricemia					
No	65(61.9)	25(23.8)	9(8.6)	31(29.5)	0.023
Yes	40(38.1)	6(5.7)	11(10.5)	23(21.9)	

At the baseline, the participants were also evaluated for the biochemical parameters based on CKD stages. A statistically significant difference was observed for hemoglobin, serum creatinine, total protein levels, serum uric acid, and urea levels (*p* < 0.05). CKD: Chronic Kidney Disease.

**Table 3:** Distribution of baseline biochemical parameters based on CKD stages.

Parameter	N (105)	Stage 3 (*n* = 34)	Stage 4 (*n* = 20)	Stage 5 (*n* = 54)	*p* Value
Age	54.53 ± 13.47	51.83 ± 14.87	57.25 ± 10.90	55.07 ± 13.45	0.346
GFR (mL/min/1.73 m^2^)	21.01 ± 16.70	44.38 ± 7.81	20.75 ± 3.98	7.68 ± 2.66	<0.05
HB (g/dL)	8.87 ± 2.12	10.35 ± 1.75	9.72 ± 1.90	7.70 ± 1.69	<0.05
S. Creatinine (mg/dL)	4.99 ± 3.32	1.65 ± 0.29	3.05 ± 0.72	7.63 ± 2.52	<0.05
Sodium (mg/dL)	135.16 ± 6.57	134.25 ± 7.62	136.25 ± 5.29	135.27 ± 6.57	0.567
Total Protein (g/dL)	5.87 ± 1.15	5.19 ± 1.19	5.24 ± 1.12	6.11 ± 0.97	<0.05
Chloride (mg/dL)	100.20 ± 11.29	97.99 ± 18.42	101.00 ± 5.70	101.17 ± 6.51	0.410
Potassium (mg/dL)	4.97 ± 3.76	4.36 ± 0.83	4.70 ± 0.84	5.43 ± 5.16	0.426
Albumin (g/dL)	3.25 ± 0.69	2.98 ± 0.89	3.26 ± 0.67	3.39 ± 0.52	0.030
S. Uric acid (mg/dL)	7.30 ± 7.06	5.58 ± 1.64	8.30 ± 3.53	8.92 ± 9.45	<0.05
S. Urea (mg/dL)	118.17 ± 81.07	57.54 ± 41.06	87.70 ± 39.26	164.26 ± 82.01	<0.05

CKD: Chronic Kidney Disease; HB: Hemoglobin; GFR: Glomerular Filtration Rate; S: Serum.

**Table 4:** HRQoL dimension scores in different stages of CKD.

HRQoL Dimensions	Total (*n* = 105)	CKD Stage 3 (*n* = 34)	CKD Stage 4 (*n* = 20)	CKD Stage 5 (*n* = 54)	*p* Value
Symptom problem list	67.10 ± 15.23	88.57 ± 4.12	59.27 ± 6.02	57.71 ± 7.22	<0.005
Effect of kidney disease	44.08 ± 23.85	78.45 ± 4.65	39.99 ± 11.38	25.85 ± 6.96	<0.005
The burden of kidney disease	30.23 ± 19.49	48.80 ± 21.61	45.62 ± 16.61	35.76 ± 12.34	<0.005
Work status	32.38 ± 37.32	59.67 ± 41.67	22.00 ± 25.13	21.29 ± 30.09	<0.005
Cognitive function	49.14 ± 32.85	91.18 ± 9.64	63.00 ± 7.93	19.87 ± 8.03	<0.005
Quality of social interaction	57.20 ± 22.02	87.95 ± 6.79	57.66 ± 6.93	19.87 ± 8.03	<0.005
Sexual function	0.000	0.000	0.000	0.000	<0.005
Sleep	49.97 ± 17.50	72.17 ± 8.84	42.12 ± 3.91	40.13 ± 11.88	<0.005
Social support	75.39 ± 23.12	100.00 ± 0.00	77.50 ± 18.94	71.60 ± 16.06	<0.005
Dialysis staff encouragement	86.07 ± 14.22	91.12 ± 9.23	85.00 ± 0.00	78.00 ± 13.93	<0.005
Overall health	42.76 ± 14.83	60.32 ± 7.06	47.00 ± 7.32	31.11 ± 7.68	<0.005
Patient satisfaction	62.53 ± 16.46	82.79 ± 10.07	58.33 ± 8.55	52.46 ± 9.93	<0.005
Physical functioning	34.85 ± 25.92	72.09 ± 7.82	29.25 ± 9.21	15.55 ± 7.18	<0.005
Role of physical health	16.66 ± 31.52	47.41 ± 43.94	45.00 ± 23.78	0.00 ± 0.00	<0.005
Pain	37.38 ± 34.25	87.01 ± 9.75	32.00 ± 8.41	10.87 ± 7.46	<0.005
General health	32.90 ± 15.28	54.83 ± 5.84	25.75 ± 5.68	22.96 ± 5.09	<0.001
Emotional well-being	38.89 ± 28.25	77.16 ± 6.21	46.80 ± 6.63	14.00 ± 4.23	<0.001
Role of emotional health	31.11 ± 28.22	56.99 ± 15.38	53.33 ± 16.75	8.02 ± 14.38	<0.001
Social functioning	35.83 ± 32.98	79.83 ± 8.34	44.37 ± 10.31	7.40 ± 8.59	<0.001
Energy fatigue	37.57 ± 7.30	43.38 ± 5.68	40.00 ± 6.99	32.96 ± 4.80	<0.001
SF12 PHYSICAL COMPOSITE	31.77 ± 8.40	42.02 ± 6.45	32.05 ± 4.70	25.78 ± 3.02	<0.001
SF2 MENTAL COMPOSITE	35.40 ± 10.89	47.94 ± 3.59	40.24 ± 4.63	25.70 ± 4.63	<0.001

CKD: Chronic Kidney Disease; SF: Short-Form; HRQoL: Health-Related Quality of Life.

**Table 5:** Correlation between PCS and various covariates.

Parameter	Correlation Coefficient	*p* Values
Age	−0.031	0.755
GFR (mL/min/1.73 m^2^)	0.512 **	<0.005
Hb (g/dL)	0.378 **	<0.005
S. Creatinine (mg/dL)	−0.665 **	<0.005
S. Sodium (mg/dL)	−0.140	0.155
Total Protein (g/dL)	−0.257 **	0.008
S. chloride (mg/dL)	−0.256 **	0.009
S. Potassium(mg/dL)	−0.066	0.504
Albumin (g/dL)	−0.167	0.088
S. Uric acid (mg/dL)	−0.117	0.236
S. Urea (mg/dL)	−0.450 **	<0.005

** The significance level for correlation is 0.01 (2-tailed). CKD: Chronic Kidney Disease; Hb: Hemoglobin; GFR: Glomerular Filtration Rate; S: Serum; PCS: Physical Composite Summary.

**Table 6:** Correlation between MCS and various covariates.

Parameter	Correlation Coefficient	*p* Values
Age	−0.117	0.234
GFR (mL/min/1.73 m^2^)	0.836 **	<0.005
HB (g/dL)	0.488 **	<0.005
S. Creatinine (mg/dL)	−0.769 **	0.001
S. Sodium (mg/dL)	−0.042	0.673
Total Protein (g/dL)	−0.305 **	0.002
S. chloride (mg/dL)	−0.59	0.552
S. Potassium(mg/dL)	−0.162	0.099
Albumin (g/dL)	−0.279 **	0.004
S. Uric acid (mg/dL)	−0.124	0.206
S. Urea (mg/dL)	−0.640 **	<0.005

** Correlation is significant at the 0.01 level (2-tailed). CKD: Chronic Kidney Disease; Hb: Hemoglobin; GFR: Glomerular Filtration Rate; S: Serum; MCS: Mental Composite Summary.

## Data Availability

Data shall be provided on request.

## Supplementary Materials

The following supporting information can be downloaded at: https://www.mdpi.com/article/10.3390/medicina59101788/s1, questionnaire: YOUR HEALTH.
