# Supplementary material for: Assessment of Health-Related Quality of Life in Chronic Kidney Disease Patients: A Hospital-Based Cross-Sectional Study
Source: Medicina (Kaunas). 2023 Oct 8;59(10):1788. doi: 10.3390/medicina59101788 (PMC10608694; doi:10.3390/medicina59101788)
Supplement: Supplementary file 1 [file medicina-59-01788-s001.zip › medicina-2611306-supplementary.pdf]

YOUR HEALTH

1. In general, would you say your health is:

(Circle One Number)

Excellent	1
Very good	2
Good	3
Fair	4
Poor	5

2. **Compared to one year ago**, how would you rate your health in general **now**?

(Circle One Number)

Much better now than one year ago	1
Somewhat better now than one year ago	2
About the same as one year ago	3
Somewhat worse now than one year ago	4
Much worse now than one year ago	5

Copyright © RAND 1995, 1996, 1997

3. The following items are about activities you might do during a typical day. **Does your health now limit** you in these activities? If so, how much?

(Circle One Number on Each Line)

	Yes, Limited a Lot	Yes, Limited a Little	No, Not Limited at All
a. **Vigorous activities**, such as running, lifting heavy objects, participating in strenuous sports	1	2	3
b. **Moderate activities**, such as moving a table, pushing a vacuum cleaner, bowling, or playing golf	1	2	3
c. Lifting or carrying groceries	1	2	3
d. Climbing **several** flights of stairs ...	1	2	3
e. Climbing **one** flight of stairs	1	2	3
f. Bending, kneeling, or stooping	1	2	3
g. Walking **more than a mile**	1	2	3
h. Walking **several blocks**	1	2	3
i. Walking **one block**	1	2	3
j. Bathing or dressing yourself	1	2	3

 Copyright © RAND 1995, 1996, 1997

4. During the **past 4 weeks**, have you had any of the following problems with your work or other regular activities **as a result of your physical health?**

(Circle One Number on Each Line)

	Yes	No
a. Cut down the **amount of time** you spent on work or other activities?	1	2
b. **Accomplished less** than you would have liked?........	1	2
c. Were limited in the **kind** of work or other activities?.....	1	2
d. Had **difficulty** performing the work or other activities (for example, it took extra effort)?	1	2

5. During the **past 4 weeks,** have you had any of the following problems with your work or other regular daily activities **as a result of any emotional problems** (such as feeling depressed or anxious)?

(Circle One Number on Each Line)

	Yes	No
a. Cut down the **amount of time** you spent on work or other activities?	1	2
b. **Accomplished less** than you would like?	1	2
c. Didn't do work or other activities **as carefully as usual?**	1	2

6. During the **past 4 weeks**, to what **extent** have your **physical health or emotional problems** interfered with your normal social activities with family, friends, neighbors, or groups?

(Circle One Number)

Not at all	1
Slightly	2
Moderately	3
Quite a bit	4
Extremely	5

7. How much **bodily** pain have you had during the **past 4 weeks?**

(Circle One Number)

None	1
Very mild	2
Mild	3
Moderate	4
Severe	5
Very severe	6

8. During the **past 4 weeks**, how much did **pain** interfere with your normal work (including both work outside the home and housework)?

(Circle One Number)

Not at all	1
A little bit	2
Moderately	3
Quite a bit	4
Extremely	5

 Copyright © RAND 1995, 1996, 1997

9. These questions are about how you feel and how things have been with you **during the past 4 weeks.** For each question, please give the one answer that comes closest to the way you have been feeling.

How much of the time during the **past 4 weeks** . . .

(Circle One Number on Each Line)

	All of the Time	Most of the Time	A Good Bit of the Time	Some of the Time	A Little of the Time	None of the Time
a. Did you feel full of pep?	1	2	3	4	5	6
b. Have you been a very nervous person?	1	2	3	4	5	6
c. Have you felt so down in the dumps that nothing could cheer you up?	1	2	3	4	5	6
d. Have you felt calm and peaceful?	1	2	3	4	5	6
e. Did you have a lot of energy?	1	2	3	4	5	6
f. Have you felt downhearted and blue?	1	2	3	4	5	6
g. Did you feel worn out?	1	2	3	4	5	6
h. Have you been a happy person?	1	2	3	4	5	6
i. Did you feel tired?	1	2	3	4	5	6

 Copyright © RAND 1995, 1996, 1997

10. During the **past 4 weeks,** how much of the **time** have your **physical health or emotional problems** interfered with your social activities (like visiting with friends, relatives, etc.)?

(Circle One Number)

All of the time	1
Most of the time	2
Some of the time	3
A little of the time	4
None of the time	5

11. Please choose the answer that best describes how **TRUE** or **FALSE** each of the following statements is for you.

(Circle One Number on Each Line)

	Definitely True	Mostly True	Don't Know	Mostly False	Definitely False
a. I seem to get sick a little easier than other people	1	2	3	4	5
b. I am as healthy as anybody I know	1	2	3	4	5
c. I expect my health to get worse	1	2	3	4	5
d. My health is excellent	1	2	3	4	5

 Copyright © RAND 1995, 1996, 1997

YOUR KIDNEY DISEASE

12. How **TRUE** or **FALSE** is each of the following statements for you?

(Circle One Number on Each Line)

	Definitely True	Mostly True	Don't Know	Mostly False	Definitely False
a. My kidney disease interferes too much with my life	1	2	3	4	5
b. Too much of my time is spent dealing with my kidney disease	1	2	3	4	5
c. I feel frustrated dealing with my kidney disease	1	2	3	4	5
d. I feel like a burden on my family	1	2	3	4	5

Copyright © RAND 1995, 1996, 1997

13. These questions are about how you feel and how things have been going during the **past 4 weeks.** For each question, please give the one answer that comes closest to the way you have been feeling.

How much of the time during the **past 4 weeks** . . .

(Circle One Number on Each Line)

	None of the Time	A Little of the Time	Some of the Time	A Good bit of the Time	Most of the Time	All of the Time
a. Did you isolate yourself from people around you?	1	2	3	4	5	6
b. Did you react slowly to things that were said or done?	1	2	3	4	5	6
c. Did you act irritable toward those around you?	1	2	3	4	5	6
d. Did you have difficulty concentrating or thinking?	1	2	3	4	5	6
e. Did you get along well with other people?	1	2	3	4	5	6
f. Did you become confused?	1	2	3	4	5	6

 Copyright © RAND 1995, 1996, 1997

14. During the **past 4 weeks,** to what extent were you bothered by each of the following?

(Circle One Number on Each Line)

	Not at All bothered	Somewhat bothered	Moderately bothered	Very Much bothered	Extremely bothered
a. Soreness in your muscles?...............	1	2	3	4	5
b. Chest pain?...........	1	2	3	4	5
c. Cramps?	1	2	3	4	5
d. Itchy skin?	1	2	3	4	5
e. Dry skin?...............	1	2	3	4	5
f. Shortness of breath?..................	1	2	3	4	5
g. Faintness or dizziness?	1	2	3	4	5
h. Lack of appetite?	1	2	3	4	5
i. Washed out or drained?................	1	2	3	4	5
j. Numbness in hands or feet?.......	1	2	3	4	5
k. Nausea or upset stomach?	1	2	3	4	5

Copyright © RAND 1995, 1996, 1997

14. (Continued) During the **past 4 weeks,** to what extent were you bothered by each of the following?

(Circle One Number on Each Line)

	Not at All bothered	Somewhat bothered	Moderately bothered	Very Much bothered	Extremely bothered
Hemodialysis Patient only					
l. Problems with your access site?...............	1	2	3	4	5
Peritoneal Dialysis Patient only					
m. Problems with your catheter site?.............	1	2	3	4	5

Copyright © RAND 1995, 1996, 1997

EFFECTS OF KIDNEY DISEASE ON YOUR DAILY LIFE

15. Some people are bothered by the effects of kidney disease on their daily life, while others are not. How much does kidney disease **bother** you in each of the following areas?

(Circle One Number on Each Line)

	Not at All bothered	Somewhat bothered	Moderately bothered	Very Much bothered	Extremely bothered
a. Fluid restriction?	1	2	3	4	5
b. Dietary restriction?	1	2	3	4	5
c. Your ability to work around the house?	1	2	3	4	5
d. Your ability to travel?	1	2	3	4	5
e. Being dependent on doctors and other medical staff?	1	2	3	4	5
f. Stress or worries caused by kidney disease?................	1	2	3	4	5
g. Your sex life?........	1	2	3	4	5
h. Your personal appearance?	1	2	3	4	5

 Copyright © RAND 1995, 1996, 1997

The next three questions are personal and relate to your sexual activity, but your answers are important in understanding how kidney disease impacts on people's lives.

16. Have you had any sexual activity in the **past 4 weeks?**

(Circle One Number)

No .... 1 ---> Please skip to Question 17

Yes 2

⬇

How much of a problem was each of the following in the **past 4 weeks?**

(Circle One Number on Each Line)

	Not a Problem	A Little Problem	Somewhat of a Problem	Very Much a Problem	Severe Problem
a. Enjoying sex?	1	2	3	4	5
b. Becoming sexually aroused?	1	2	3	4	5

For the following question, please rate your sleep using a scale ranging from 0 representing "very bad" to 10 representing "very good."

If you think your sleep is half-way between "very bad" and "very good," please circle 5. If you think your sleep is one level better than 5, circle 6. If you think your sleep is one level worse than 5, circle 4 (and so on).

17. On a scale from **0** to **10**, how would you rate your sleep overall?

(Circle One Number)

0 1 2 3 4 5 6 7 8 9 10

Very Bad — Very Good

18. How often during the **past 4 weeks** did you...

(Circle One Number on Each Line)

	None of the Time	A Little of the Time	Some of the Time	A Good bit of the Time	Most of the Time	All of the Time
a. Awaken during the night and have trouble falling asleep again?	1	2	3	4	5	6
b. Get the amount of sleep you need?	1	2	3	4	5	6
c. Have trouble staying awake during the day?	1	2	3	4	5	6

19. Concerning your **family and friends,** how satisfied are you with ...

(Circle One Number on Each Line)

	Very Dissatisfied	Somewhat Dissatisfied	Somewhat Satisfied	Very Satisfied
a. The amount of time you are able to spend with your family and friends?......................	1	2	3	4
b. The support you receive from your family and friends?.....	1	2	3	4

 Copyright © RAND 1995, 1996, 1997

20. During the **past 4 weeks,** did you work at a paying job?

(Circle One Number)

Yes	1
No	2

21. Does your health keep you from working at a paying job?

(Circle One Number)

Yes	1
No	2

22. Overall, how would you rate your health?

(Circle One Number)

0	1	2	3	4	5	6	7	8	9	10
Worst possible (as bad or worse than being dead)					Half-way between worst and best					Best Possible Health

Copyright © RAND 1995, 1996, 1997

SATISFACTION WITH CARE

23. Think about the care you receive for kidney dialysis. In terms of your satisfaction, how would you rate the friendliness and interest shown in you as a person?

(Circle One Number)

Very Poor	1
Poor	2
Fair	3
Good	4
Very Good	5
Excellent	6
The Best	7

24. How **TRUE** or **FALSE** is each of the following statements?

(Circle One Number on Each Line)

	Definitely True	Mostly True	Don't Know	Mostly False	Definitely False
a. Dialysis staff encourage me to be as independent as possible	1	2	3	4	5
b. Dialysis staff support me in coping with my kidney disease	1	2	3	4	5

 Copyright © RAND 1995, 1996, 1997

BACKGROUND INFORMATION

25. Do you currently take prescription medications regularly (4 or more days a week) that are prescribed by your doctor for a medical condition? Please don't count over the counter medications like antacids or aspirin.

(Circle One Number)

No .... 1 ---> Please skip to Question 26

Yes 2

⬇

25a. How many different prescription medications do you currently take?

Number of Medications: ________

26. How many days total in the **last 6 months** did you stay in any hospital overnight or longer? **(If none, please write in 0)**

Number of Days: ________

27. How many days total in the **last 6 months** did you receive care at a hospital, but came home the same day? (**If none, please write in 0**)

Number of Days: ________

 Copyright © RAND 1995, 1996, 1997

28. What caused your kidney disease?

(Circle All That Apply)

Don't know	1
Hypertension (High Blood Pressure)	2
Diabetes	3
Polycystic Kidney Disease	4
Chronic Glomerulonephritis	5
Chronic Pyelonephritis	6
Other (please specify): ______________________	7

____

29. When were you born?

[ ][ ] / [ ][ ] / [ ][ ]
Month / Day / Year

30. What is the highest level of school you have completed?

(Circle One Number)

8th grade or less	1
Some high school or less	2
High school diploma or GED...............................	3
Vocational school or some college	4
College degree	5
Professional or graduate degree	6

 Copyright © RAND 1995, 1996, 1997

31. What is your gender?

(Circle One Number)

Male 1

Female 2

32. How do you describe yourself?

(Circle One Number)

African American or Black 1

Hispanic or Latino 2

Native American or American Indian 3

Asian or Pacific Islander 4

White 5

Other (please specify): ____________________ 6

____

33. Are you currently married?

(Circle One Number)

No 1

Yes 2

 Copyright © RAND 1995, 1996, 1997

34. During the **last 30 days,** were you:

(Circle One Number)

Working full-time	1
Working part-time	2
Unemployed, laid off, or looking for work	3
Retired	4
Disabled	5
In school	6
Keeping house	7
None of the above	8

35. What kind of health insurance do you have?

(Circle One Number)

None, I have no health insurance	1
Medicare only	2
Medicare and any other insurance	3
Medicaid or Medi-Cal only	4
Private, fee-for-service health insurance (e.g., Prudential, Aetna, etc.)	5
HMO, PPO, IPA or other prepaid plan (e.g., Kaiser, Cigna, FHP, etc.)	6
Other (please specify)	7

____

____

 Copyright © RAND 1995, 1996, 1997

36. What was your total household income (from all sources) before taxes in the **LAST CALENDAR YEAR**, including yourself, your partner, and others you regard as family who live in your household? (Please remember your answers are confidential.)

(Circle One Number)

Less than $5,000	1
$5,001-$10,000	2
$10,001-$20,000	3
$20,001-$40,000	4
$40,001-$75,000	5
More than $75,000	6
Don't know	7

37. Did someone help you fill out this survey?

(Circle One Number)

Yes, a physician or other health care provider	1
Yes, a family member or friend	2
Yes, someone else	3
No	4

38. What is today's date?

__ __	/	__ __	/	__ __
Month		Day		Year

THANK YOU FOR TAKING PART IN THIS STUDY.

Copyright © RAND 1995, 1996, 1997
